# Ecological divergence and evolutionary transition of resprouting types in *Banksia attenuata*

**DOI:** 10.1002/ece3.1143

**Published:** 2014-07-22

**Authors:** Tianhua He

**Affiliations:** Department of Environment and Agriculture, Curtin UniversityPO Box U1987, Perth, WA, 6845, Australia

**Keywords:** Epicormic, evolutionary transition, genetic differentiation, lignotuber, morphological divergence, resprouting

## Abstract

Resprouting is a key functional trait that allows plants to survive diverse disturbances. The fitness benefits associated with resprouting include a rapid return to adult growth, early flowering, and setting seed. The resprouting responses observed following fire are varied, as are the ecological outcomes. Understanding the ecological divergence and evolutionary pathways of different resprouting types and how the environment and genetics interact to drive such morphological evolution represents an important, but under-studied, topic. In the present study, microsatellite markers and microevolutionary approaches were used to better understand: (1) whether genetic differentiation is related to morphological divergence among resprouting types and if so, whether there are any specific genetic variations associated with morphological divergence and (2) the evolutionary pathway of the transitions between two resprouting types in *Banksia attenuata* (epicormic resprouting from aerial stems or branch; resprouting from a underground lignotuber). The results revealed an association between population genetic differentiation and the morphological divergence of postfire resprouting types in *B. attenuata*. A microsatellite allele has been shown to be associated with epicormic populations. Approximate Bayesian Computation analysis revealed a likely evolutionary transition from epicormic to lignotuberous resprouting in *B. attenuata*. It is concluded that the postfire resprouting type in *B. attenuata* is likely determined by the fire's characteristics. The differentiated expression of postfire resprouting types in different environments is likely a consequence of local genetic adaptation. The capacity to shift the postfire resprouting type to adapt to diverse fire regimes is most likely the key factor explaining why *B. attenuata* is the most widespread member of the *Banksia* genus.

## Introduction

Plants resprout from aboveground or subterranean buds following damage to or the death of their aboveground tissue. Resprouting is a key functional trait that confers persistence and enables plants to survive diverse disturbance regimes. As a consequence, the plant community is resilient to severe disturbances, for example, fire and drought (Clarke et al. 2013). In fire-prone ecosystems, plant species that are capable of resprouting after their aboveground biomass is damaged or killed by fire constitute 50–100% of the flora (Williams et al. 2002; Pausas et al. 2006; Enright et al. 2007). Apart from the presence of stored resources such as starch and nutrients (Moreira et al. 2012), a crucial morphological feature for successful resprouting is the presence of buds or meristems located in plant parts sufficiently insulated from the lethal heat of the fire (Lamont et al. 2011). Therefore, a plant can resprout either epicormically (resprouting from aerial stems or branch) or through a lignotuber (a woody swelling of the root crown) or root (Bond and Midgley 2003; Clarke et al. 2013). Much research has been conducted on the ecology of the two contrasting postfire regeneration strategies, that is, seeding and resprouting (e.g., Pausas and Verdu′ 2005; Verdu′ et al. 2007; Segarra-Moragues and Ojeda 2010), whereas research addressing the evolution of different types of postfire resprouting responses (Lamont et al. 2013). Understanding the divergence of postfire resprouting strategies requires knowledge of the causes of divergence, their relative importance, and how they interact to form the environment-genetics landscape.

Resprouting, for example the ability to form a lignotuber, is genetically controlled, whereas the expression of that ability may depend on local circumstances (Whittock et al. 2003; Verdaguer and Ojeda 2005; Groom and Lamont 2011; Clarke et al. 2013). Because of this, variations in resprouting types among populations are common. For example, at least five *Banksia* spp. exhibit both epicormic and lignotuberous resprouting in different populations in fire-prone environments (Taylor and Hopper 1988; Collins et al. 2008; Groom and Lamont 2011). It has also been reported that populations of some banksias resprout epicormically in less stressful environments, whereas lignotubers form in more stressful environments (Cowling and Lamont 1985; Groom and Lamont 2011). However, it is not clear whether the divergence in resprouting behavior is driven by genetic changes. Ecologically relevant genetic variation can be explored by searching genes or through locus marker-associated selection. Loci that show unusually strong associations with environmental variables may be under selection that is driven by those environmental factors or associated selection pressures (Coop et al. 2010). The studies that have been performed to date on ecologically relevant genetic variation are largely restricted to model organisms. Thus, it is essential to expand this work beyond model organisms to obtain a better representation of plant diversity, genetic variation, and responses to the environment (Karrenberg and Widmer 2008).

Resprouting has been proposed to improve fitness in fire-prone environments (Keeley et al. 2011; Lamont et al. 2011). Lamont et al. (2011) noted seven fitness benefits of postfire resprouting that center around the rapid return to adult growth rates, early flowering, and seed setting, without the risk of recruitment failure. For instance, epicormic resprouting after a fire could provide a competitive advantage due to the rapid re-establishment of the leaf area over the full extent of the stem and branches, resulting in more efficient sunlight interception (Dietze and Clark 2008; Waters et al. 2010). Groom and Lamont (2011) found that the adaptive response of lignotuberous populations after a fire is to produce more reproductive shoots, which is favored architecturally by the many strong stems arising directly from the lignotubers following such events. This characteristic has major fitness implications in fire-prone and water-/nutrient-limited environments, where seed availability constrains population viability, even among resprouters (Enright et al. 1998). Recent studies on the evolution of resprouting in fire-prone systems have shown that the various types of resprouting (e.g., via clonality, or epicormic bud strands) are derived from surrounding parental lineages lacking these traits (Crisp et al. 2011; He et al. 2011). This finding highlights that resprouting should not be treated as just one trait in attempting to understand its evolution. The evolutionary transitions between resprouting types and the influence of fire characteristics and the environment are intriguing; however, it remains unclear how transitions between postfire resprouting types occur.

One of the challenges in determining such transitional pathways in phenotype evolution lies in determining which population is in the ancestral state. The coalescence of alleles can provide evidence of which population bearing a particular phenotype is ancestral. Analyses of genetic variation to estimate historical parameters and to quantitatively compare alternative scenarios have recently become feasible through approximate Bayesian computation (ABC, Csilléry et al. 2010). In ABC, millions of genealogies are simulated assuming different parameter values under different models, and the simulations that produce genetic variation patterns which are close to the observed data are retained and analyzed in detail (Cornuet et al. 2008). Another advantage of ABC analysis is that unsampled populations can be included in the model, which is particularly useful if they are potentially relevant for the sampled populations, such as a population that is ancestral to the current sampled population.

In comparison to the large amount of ecological information supporting the role of natural selection as the main cause of morphological divergence and population genetic differentiation, the associations and interactions between these processes are significantly less well studied (Bernatchez et al. 2010). Microsatellite DNA markers have long been used to identify the effects of life history traits, phylogeographic history, and environmental factors on the genetic structure of plant populations. In this study, I used polymorphic microsatellite DNA markers and a microevolutionary approach to study: (1) whether genetic differentiation is related to the morphological divergence in resprouting types, and if so, whether there are any specific genetic variations associated with morphological divergence; (2) how environmental and ecological factors have shaped the patterns of genetic differentiation and morphological divergence; and (3) the evolutionary pathway of transitions in morphological characters. I focused on the species *Banksia attenuata* (Proteaceae), which includes distinct lignotuberous and epicormic populations with marked morphological divergences in a speciose southwest Australian (SWA) ecosystem (Cowling and Lamont 1985; Taylor and Hopper 1988); thus, possible confounding effects resulting from independent phylogenetic histories were avoided. Specifically, I analyzed the morphological divergence, genetic differentiation, putative selection, and gene coalescence of both lignotuberous and epicormic populations of this species using microsatellite markers to answer the above questions.

## Materials and Methods

### Study species

**Figure 1:**
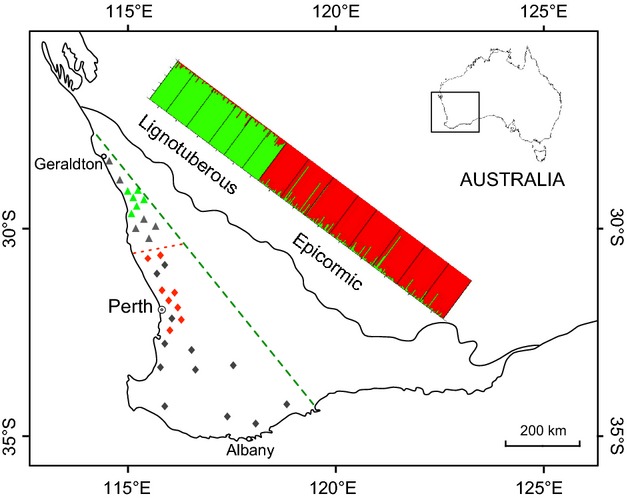
Map of the studied populations relative to the distribution of *Banksia attenuata* and concordance with Bayesian clustering analysis. The area west of the green dotted line corresponds to the approximate range of the species; the red dotted line is the suggested boundary between the growth forms (Cowling and Lamont 1985; Taylor and Hopper 1988). Gray markers represent population surveyed, colored markers represent population genotyped. Gray curve indicates rough 300-mm isohyet which defines the east boundary of South Western Australia Floristic Region.

*Banksia attenuata* is an outcrossing shrub/tree species endemic to SW Australia. It shows the widest distribution of any *Banksia* species in the region, extending from 500 km north to 300 km south and 450 km southeast of Perth, Western Australia (Taylor and Hopper 1988) (Fig. 1). *Banksia attenuata* resprouts after fire via epicormic or lignotuberous buds (in tree or shrub form, respectively). There is a marked demarcation between the two forms, with northern populations being lignotuberous and southern populations being epicormic (Taylor and Hopper 1988) (Fig. 1). Although it has been reported that both forms occur in the vicinity of the Hill River (Cowling and Lamont 1985), a recent extensive field survey failed to locate any populations in which both forms were present. The seeds of *B. attenuata* are stored on the plant for 5 years or more (serotiny), and seed release is stimulated by fire (Enright and Lamont 1989). Although the majority of the seeds are principally gravity-dispersed postfire, up to 6% of the seeds could be dispersed to distant habitats at least 2.6 km away by a wind vortex after a fire (He et al. 2009). *Banksia attenuata* is pollinated by nectar-feeding birds (predominantly honeyeaters of the Meliphagidae family), small marsupials and, to a lesser extent, bees and wasps (Collins and Rebelo 1987). *Banksia attenuata* forms an important component of open *Eucalyptus* or *Banksia* woodlands as a dominant or understory tree or tall shrub. The annual rainfall within its distributional range varies from 400 to 1000 mm.

### Morphology survey

A total of 100 individuals from at least five locations were surveyed for each of the resprouting forms (epicormic and lignotuberous) and examined regarding the following phenotypes: the presence of a lignotuber; the number of stems/trunks emerging from the ground; the height of the tallest stem/trunk; and the perimeter of the largest stem/trunk at 50 cm above the ground (many mature lignotuberous individuals are just over 100 cm in height). To confirm whether the revealed pattern of divergence in postfire resprouting type is consistent across the species' range, an extensive survey was conducted, and resprouting type (epicormic or lignotuberous) was recorded for additional 17 populations throughout the species' distribution (Fig. 1).

### Sampling and microsatellite genotyping

Healthy, intact leaves of *B. attenuata* were collected from 25 to 35 individuals from each of 14 populations: six populations in the northern part of the species' distribution and eight in the southern part, separated by the Hill River, which is suggested to be where the two forms meet (Fig. 1). Genomic DNA was extracted following the protocol described in He et al. (2004). The quantity and quality of the DNA were assessed via visualization on a 1.5% agarose gel using a spectrophotometer (NanoDrop, ND-1000, Wilmington, DE). Eleven pairs of unlinked microsatellite primers were used, and the protocol for genotyping with these primers is described in He et al. (2007).

### Genetic diversity and genetic structure

Parameters related to genetic diversity, including the mean number of alleles per locus (*A*), mean effective number of alleles per locus (*A*_E_) and observed (*H*_O_) and unbiased expected (*H*_E_) heterozygosities, were calculated for each population using GenoDive (Meirmans and van Tienderen 2004). Pairwise population differentiation estimates were characterized based on the standardized coefficient of differentiation, *F*'_ST_ (Hedrick 2005). Because the traditional *F*_ST_ is likely to underestimate genetic differentiation between populations for markers that show high levels of allelic variability, such as microsatellite DNA markers, the following standardized form of the genetic differentiation parameter was used: *F*'_ST_ = *F*_ST_/*F*_ST_ _max_ (Hedrick 2005). *F*'_ST_ was calculated and analyzed in GenoDive (Meirmans and van Tienderen 2004). Allelic differentiation at each locus was further measured using Jost's D (Jost 2008), implemented in diveRsity online (Keenan et al. 2013). Jost's D was calculated within lignotuberous populations, epicormic populations, and between lignotuberous and epicormic populations.

The population genetic structure of the plants was further investigated through a Bayesian clustering procedure implemented in STRUCTURE V.2.0 (Pritchard et al. 2000; Pritchard 2002). The Bayesian clustering procedure finds the optimal number of genetic clusters (*K*) and assigns individuals to the different clusters based on the allele frequencies at each locus. An admixture model was employed in the analysis. *K* values ranging from 2 to the total number of populations (i.e., 14) were tested. The burn-in period and run length of the Monte Carlo Markov Chain (MCMC) were 1 × 10^5^ and 1 × 10^8^ iterations, respectively. For each K, five runs were conducted. The optimum number of clusters (*K*) present in the dataset was evaluated according to Evanno et al. (2005).

### Identification of microsatellite loci associated with selection

Eleven microsatellite loci were tested for outliers (i.e., markers potentially associated with natural selection) using the program FDIST2 (Beaumont and Nichols 1996). Briefly, the null distribution of target *F*_ST_ values that would be expected from a neutral model was generated, and the 99% quantile limits were calculated. Loci outside the 99% confidence interval were regarded as potentially associated with selection. Because allele frequencies are typically correlated among closely related populations, and geographically proximate populations often share environmental variables, neighboring populations can rarely be treated as independent observations (Coop et al. 2010). Therefore, testing for selection was implemented in three sets of populations: the whole population set, the set of lignotuberous populations, and the set of epicormic populations. Loci showing signs of linkage to genes under selection in the whole population set, but not in the two separate population sets, were assumed to be associated with a postfire resprouting type.

### Evolutionary transition of resprouting types

The evolutionary transition of the resprouting types was inferred from an approximate Bayesian computation in which evolutionary scenarios were compared through posterior probabilities (Cornuet et al. 2008). Because two genetic clusters were identified associated with individuals/populations corresponding to the postfire resprouting types (see Results), three evolutionary scenarios were defined and tested: lignotuberous populations are ancestral to epicormic populations (Scenario 1); epicormic populations are ancestral (Scenario 2); and both lignotuberous and epicormic populations are descendants of an ancestral population that is not included in the study (Scenario 3). Comparison of the scenarios was implemented in the *DIYABC* software program (Cornuet et al. 2008). In each scenario, the effective population size of the ancestral population was set as a uniform prior distribution at 10,000–100,000. The derived population, with size of 10,000–100,000 and a uniform prior distribution, was presumed to have experienced a bottleneck (population size from 10 to 100, a uniform distribution) at the time (*t*_1_) of divergence. The time period of bottleneck was assumed to a uniform distribution of 250–750 years, equivalent to 1–3 generations given a generation time of 250 years for *Banksia attenuate* (He et al. 2009). The divergence time (*t*_1_) was set at average of 25,000 years before present with a standard deviation of 5000 years in a normal distribution, given that the possible age of sand dunes where lignotuberous populations were situated was estimated at 19,000–31,000 years (Krauss et al. 2006). A realistic mutation model based on a generalized stepwise model (Fu and Chakraborty 1998; Estoup et al. 2002) with a possible single nucleotide insertion/deletion mutation was chosen in the analysis. A mutation rate with 1 × 10^−4^ minimum and 1 × 10^−3^ maximum was assumed (microsatellite mutation rate was 4.4 × 10^−3^, T. He, unpubl. data). A reference table with 6 × 10^6^ simulated datasets (2 × 10^6^ for each of the three scenarios) was generated. The scenario selected as being most likely to explain the genetic data was the one with the highest posterior probabilities in 1500 of the closest datasets, which was cross-validated via logistic regression computed from 15,000 of the closest datasets. The confidence of the choice of scenario was evaluated following Cornuet et al. (2008).

## Results

### Morphological divergence and environmental variation

**Figure 2:**
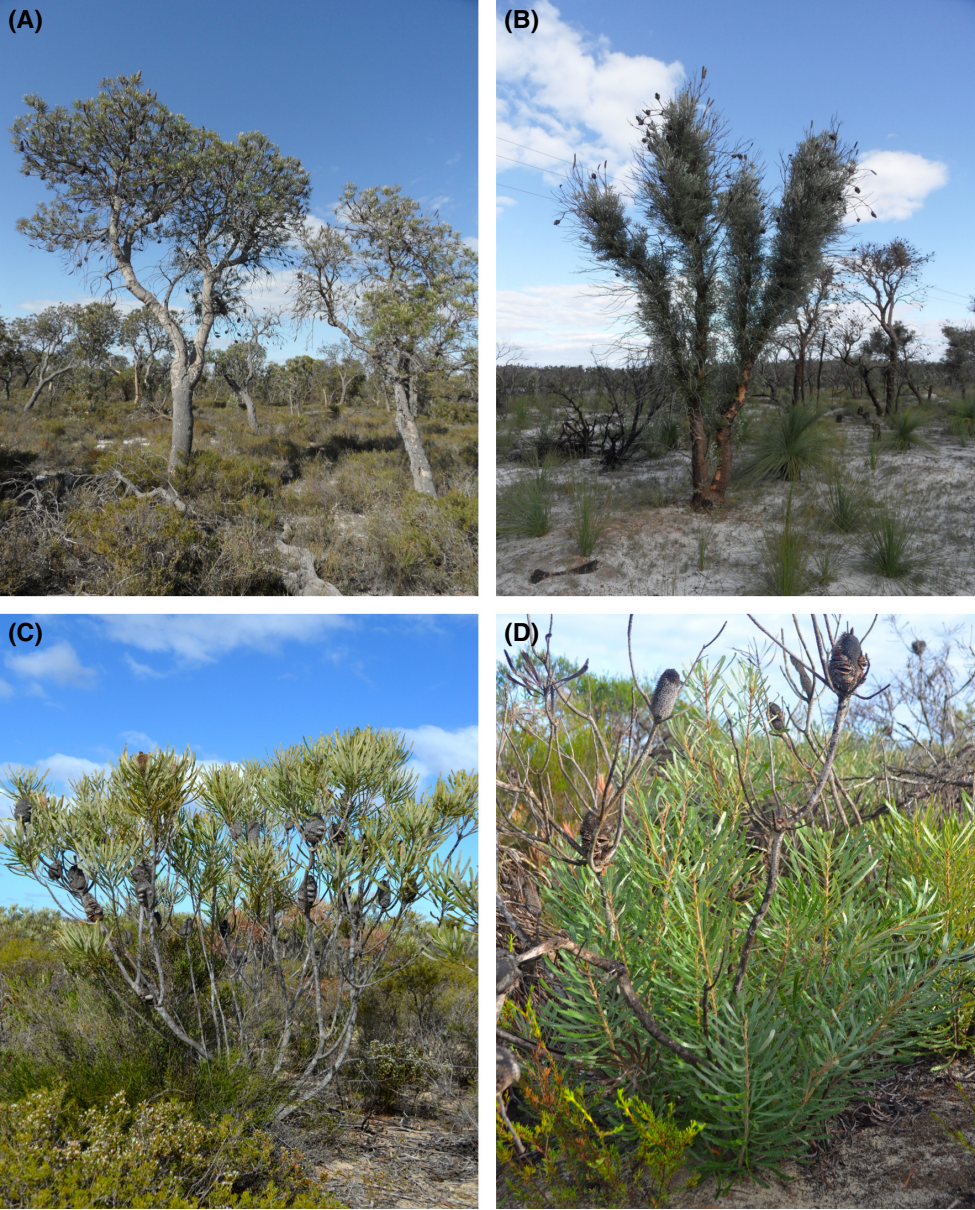
The distinctive morphologies of the two postfire resprouting types of *Banksia attenuata*. (A) Tree form (8.6 m in the figure); (B) epicormic resprouting (1 year after fire); (C) shrub form (1.8 m in the figure); (D) lignotuberous resprouting (1 year after fire).

The examined populations of *Banksia attenuata* displayed clear morphological discontinuity across the species' range (Table 1, Fig. 1). The natural populations of *B. attenuata* in the northern part of its range are exclusively lignotuberous, with a lignotuber structure being observed in all individuals. The lignotuberous individuals are multistemmed, with short (less than 2.4 m), narrow stems (smaller than 26 cm in circumference at 50 cm above the ground) (Table 1, Figs. 1, 2). In contrast, no individuals in the southern populations were found to present a lignotuberous structure. The growth form of these individuals is trees up to 10.4 m tall with one, or rarely two main trunks emerging from the ground and a circumference of up to 134 cm at 50 cm above the ground (Table 1, Figs. 1, 2). The vegetation in the northern range is heath shrubland with a canopy height of less than 2.5 m, whereas the southern sand plains are usually covered by open forest dominated by species (such as *B. attenuata*) up to 10 m tall. The northern range is drier and hotter, and its plants show a shorter growth season compared to southern part (Table 1). Extensive field surveys over the area where the two types were suggested to overlap failed to locate any mixed populations.

**Table 1:** Morphological divergence of *Banksia attenuata* and environmental variables in the two regions of its range

	Southern	Northern
Reproducing mode	Epicormic	Lignotuberous
Presence of lignotuber (%)	0	100
Canopy height (m)	8.4 ± 1.2 (2.5–10.4)	1.3 ± 0.6 (0.5–2.4)
Number of main stem	1–2	30 ± 11
Stem perimeter (cm)	80 ± 25	26 ± 12
Annual rainfall (mm)	650–1000	450–550
Temperature^1^ (monthly mean)	10–28	9–34
Growth season^1^ (months)	6.2	4.2
Fire type^1^	Surface and crown	Stand replacing
Growth type of co-occurring congeners	Tree up to 10 m	Shrub < 2.5 m

1 Cowling and Lamont (1985).

### Genetic differentiation and selection

The 11 microsatellite loci amplified a total of 198 alleles across the 14 analyzed populations. These markers revealed a slightly higher level of genetic variation in the northern lignotuberous populations when measured as the number of alleles per locus and as heterozygosity, whereas the mean effective number of alleles per locus was the same in both growth forms (Table 2). Growth form-specific alleles (with frequencies greater than 5%) were not observed.

**Table 2:** Comparison of genetic polymorphisms between northern lignotuberous populations (L1–L6) and southern epicormic populations (E1–E8) of *Banksia attenuata*. The parameters for the polymorphisms were calculated based on 25 individuals from each population

Population	*A*	*A*e	*H*o	*H*s
L1	8.4	5.2	0.63	0.76
L2	8.5	4.0	0.59	0.72
L3	8.5	4.9	0.59	0.77
L4	8.9	5.0	0.65	0.77
L5	8.4	4.2	0.64	0.73
L6	8.0	4.7	0.62	0.76
Mean	8.5 ± 0.3	4.7 ± 0.4	0.62 ± 0.02	0.75 ± 0.02
E1	7.2	4.0	0.58	0.69
E2	7.6	4.2	0.62	0.70
E3	8.1	5.0	0.57	0.74
E4	8.2	4.6	0.53	0.70
E5	8.2	4.7	0.62	0.73
E6	8.4	5.4	0.55	0.76
E7	8.0	5.0	0.54	0.71
E8	8.3	4.7	0.58	0.71
Mean	8.0 ± 0.4	4.7 ± 0.4	0.57 ± 0.03	0.72 ± 0.02

**Figure 3:**
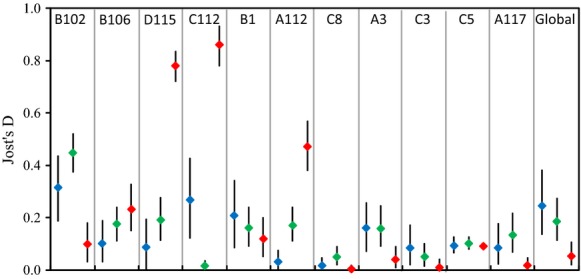
Genetic differentiation measured as Jost's D of the 11 microsatellite loci. Blue represents with differentiation within lignotuberous populations, green for within epicormic populations, red for between lignotuberous and epicormic populations. The line represents the lower and upper values generated from 1000 bootstrapping.

Genetic differentiation was not evident among the lignotuberous populations, with a mean *F*'_ST_ of −0.06 being obtained and none of the pairwise *F*'_ST_ values being significantly greater than zero (*P* < 0.05) (Table 3). A similar pattern was revealed among the epicormic populations, with a mean *F*'_ST_ of 0.03 being observed and only five (out of 28) pairs of populations being significantly differentiated (*P* < 0.05 with the Bonferroni correction). Significant genetic differentiation was revealed between the lignotuberous and epicormic populations, associated with a mean *F*'_ST_ of 0.27 (range 0.13–0.38), and all *F*'_ST_ values were significantly greater than zero (*P* < 0.05). Analysis of Jost's D revealed that the significant microsatellite genetic differentiation between the lignotuberous and epicormic populations was mainly determined by three loci. Locus D155, C112, and A112 showed much higher differentiation between the lignotuberous and epicormic populations than that of within each resprouting type (Fig. 3).

**Table 3:** Standardized coefficient of genetic differentiation (*F*'_ST_) between populations of *Banksia attenuata*

	L1	L2	L3	L4	L5	L6	E1	E2	E3	E4	E5	E6	E7
L2	−0.13												
L3	−0.09	−0.03											
L4	−0.11	−0.01	−0.05										
L5	−0.08	−0.04	0.02	−0.05									
L6	−0.14	−0.05	−0.06	−0.09	−0.06								
E1	0.23	0.31	0.29	0.28	0.33	0.29							
E2	0.19	0.28	0.27	0.25	0.29	0.26	0.01						
E3	0.16	0.27	0.26	0.28	0.30	0.24	0.01	0.04					
E4	0.20	0.31	0.33	0.27	0.28	0.23	0.10	0.08	0.11				
E5	0.22	0.29	0.30	0.29	0.29	0.27	−0.01	0.00	−0.01	0.07			
E6	0.15	0.28	0.26	0.24	0.30	0.24	0.02	0.03	−0.05	0.13	−0.01		
E7	0.13	0.25	0.28	0.32	0.36	0.30	−0.02	0.03	0.01	0.12	−0.02	0.04	
E8	0.15	0.29	0.30	0.33	0.38	0.30	0.01	0.06	0.05	0.11	0.00	0.03	−0.05

The Bayesian clustering analysis was able to cluster individuals into populations under the assumption that *K* = 2, and the most likely number of clusters was *K* = 2. The pattern of the clustering of individuals corresponded to the growth forms, that is, generating a cluster of lignotuberous populations and a cluster of epicormic populations, except that three epicormic individuals were marginally clustered with the lignotuberous populations (Fig. 1).

**Figure 4:**
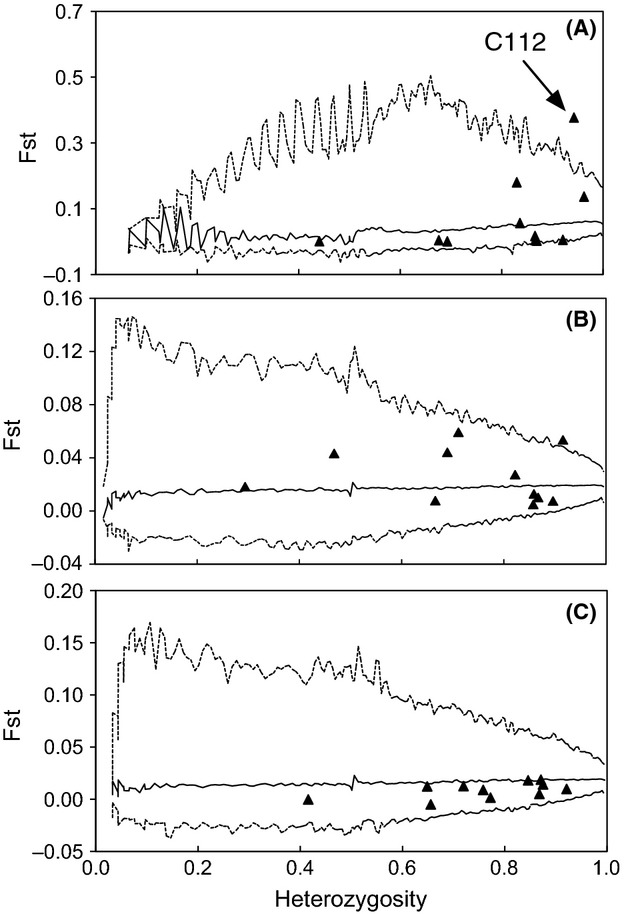
*F*_ST_ values for 11 microsatellite loci examined in *B. attenuata* populations plotted against the heterozygosity. The lines represent the median and 99% quantiles (dotted lines) of the expected *F*_ST_ values from a neutral model. (A) Analysis including all populations; (B) epicormic populations only; (C) lignotuberous populations only.

Outlier tests detected a significant departure of the *F*_ST_ value from the neutral expectation for locus BA-C112 (*F*_ST_ = 0.39, Jost's D = 0.93) in the analysis including all six lignotuberous and eight epicormic populations (Fig. 4A). However, locus BA-C112 was not an outlier in the analyses including either only the epicormic populations (*F*_ST_ = 0.018, Fig. 4B, and Jost's D = 0.02) or only the lignotuberous populations (*F*_ST_ = 0.014, Fig. 4C, and Jost's D = 0.24). Seventeen alleles were amplified at locus BA-C112 in the epicormic populations, among which allele 6 dominated, with a frequency of 0.87; no other allele exhibited a frequency greater than 0.05. The same set of alleles was revealed at BA-C112 in the lignotuberous populations, where no allele showed a frequency greater than 0.2.

**Figure 5:**
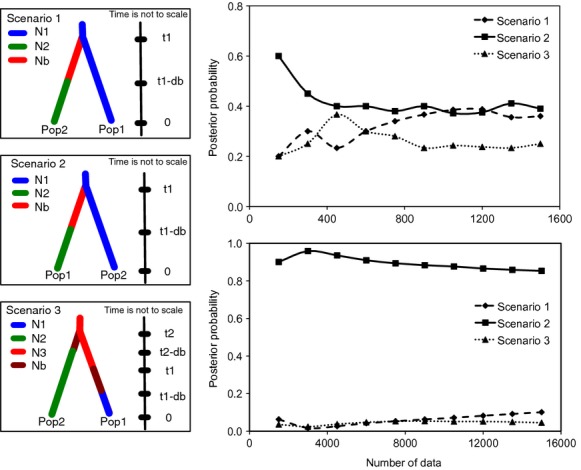
Comparison of the hypotheses for the evolutionary transition of postfire resprouting types in *Banksia attenuata*. (A) The three tested scenarios (Pop1 and N1: lignotuberous population and its effective population size; Pop2 and N2: epicormic population and its effective population size; Nb: new population with derived resprouting type; *t*_1_: time before present when new derived resprouting type first appeared; db: the time period of population bottleneck); (B) direct comparison of the posterior probabilities of the three scenarios; (C) logistic regression of the posterior probabilities of the three scenarios.

The hypotheses regarding the evolutionary transitions between the two growth forms (corresponding to the two postfire resprouting types) were tested via approximate Bayesian computation examining three scenarios (Fig 5A). The direct approach using the 1,500 closest simulated datasets showed that Scenario 2 was favored over the two other scenarios with a higher posterior probability, though the differences among the three scenarios were not clear cut (Fig 5B). Further logistic regression over the 15,000 closest datasets provided much stronger support for Scenario 2, with a consistently higher posterior probability being obtained (Fig. 5C). The confidence level for Scenario 2 being favored over the other two scenarios was 0.71. The results therefore demonstrate the evolutionary transition from epicormic resprouting to lignotuberous resprouting in *B. attenuata* during the expansion from the southern to the northern parts of its range. ABC analysis revealed that lignotuberous resprouting in the studied populations likely derived from epicormic population at 23,800 years ago (19,600–28,800, 5–95% quantile) through a short period (470 years, 410–500, 5–95% quantile) of bottleneck (with population size of 50).

## Discussion

### Ecology and adaptation of postfire resprouting types

The northern populations of *B. attenuata* were exclusively lignotuberous and showed distinctive morphological characteristics such as multiple stems and a short stature, whereas the southern populations were epicormic and presented a higher stature and usually a single stem. Among 20 resprouting *Banksia* species recorded in SWA, ten have been observed to resprout from lignotubers and/or epicormic buds and the others through rhizomes or root suckers (Taylor and Hopper 1988; Collins et al. 2008; He et al. 2011). Five *Banksia* species have the capacity to resprout from either lignotubers or epicormic buds, with their northern populations being lignotuberous and southern populations epicormic, however, co-occurrence of the two resprouting types with a population has not been found. On the other hand, the five banksias species showing both resprouting strategies are scattered throughout the genus phylogeny (He et al. 2011), it is likely there was a repeated evolution of the different strategies within each of these species. One species, *Banksia littoralis*, most commonly recorded as 10-m tall trees in seasonally wet areas and around lake margins, resprouts strictly epicormically after fire. Strictly lignotuberous species occur in areas with annual rainfall close to 300 mm (Collins et al. 2008).

Keeley et al. (2011) speculated that different resprouting types might have appeared in different lineages in response to different evolutionary pressures. This study demonstrated that different resprouting types may operate at the regional level and that local environment could have been a major force driving such morphological/ecophysiological divergence. Soil heterogeneity has been proposed as a prominent factor determining the distributions of plant species in SWA; however, no significant difference in the soil nutrient composition was observed in the southern and northern parts of the range of *B. attenuata* (Lamont 1995).

The postfire resprouting type in *B. attenuata* is likely determined by the fire's characteristics. In fire-prone ecosystems, the fire regime is influenced by factors including the fuel type, vegetation structure, and climate involved (Archibald et al. 2009). In the northern sandplain scrub-heath of SWA, wildfires are frequent with an average return time at 13 years, and are of a sufficiently high intensity to consume the crowns of all species (Miller et al. 2007).

In contrast, in the southern *Banksia* woodlands, where there is a wetter climate and higher annual rainfall, intense stand-replacing fires are rare at >50 years, while less intense mild fires are more frequent at every 3–10 years (Gill 2012). Resprouting from epicormic bud-bearing meristem stands is hypothesized to be an adaption to fire environments (Burrows 2002; Crisp et al. 2011; He et al. 2012). Epicormic stands of *Eucalyptus* showing bud-forming potential across the full thickness of the bark and outer wood allow resprouting from the stem after fire, as the thick bark provides efficient protection from the lethal heat of the fire (Burrows 2002; Waters et al. 2010). Though it has not been investigated anatomically, similar epicormic resprouting is common among Proteaceae, particularly *Banksia* species (Groom and Lamont 2011; Lamont et al. 2011). In low-intensity fires, thick bark provides efficient protection, and the hot air causes leaf scorching, but not the death of the epicormic meristems in the inner bark (Brando et al. 2012). Therefore, in wetter environments associated with low-intensity fires, epicormic resprouting after fire could provide a competitive advantage due to the rapid re-establishment of the leaf area over the full extent of the stem and branches, enabling more efficient sunlight interception and consequently shading competitors (Burrows 2002; Dietze and Clark 2008; Waters et al. 2010). In the events of stand-replacing high intense fire, at least some of deep buried bud-forming tissue are sufficiently protected by thick bark and can still regenerate (Clarke 2002).

The shorter stature of the lignotuberous populations implies that fires are more likely to reach the crown and to be more intense, with more killing power and that all of the aboveground vegetation is more likely to be killed (Groom and Lamont 2011), which effectively limits resprouting from an aboveground bud bank. A plant's lignotuber is buried in the soil as it develops, providing the plant with a bank of underground meristematic tissue that is protected from the heat of a fire (Boland et al. 1984; Noble 2001). Lignotubers therefore represent an effective mechanism for regenerating buds in habitats that frequently experience intensive fire. Strictly lignotuberous banksias occur in drier environments (Lamont and Connell 1996). However, lignotuberous resprouting is not likely to be a direct adaptation to dry habitats, as seedlings of lignotuberous species are reported to be more susceptible to drought because resource allocation for lignotuber development during the first years of growth likely reduces soil exploitation by the roots (Frazer and Davis 1988; Enright and Lamont 1992).

### Morphological divergence and genetic differentiation

It is theoretically predicted that random genetic drift in small isolated populations can lead to phenotypic divergence and genetic differentiation; however, both epicormic and lignotuberous populations of *B. attenuata* are widespread and there are no obvious ecological/physical barriers against gene exchange between the two forms. A previous study showed that *B. attenuata* seeds can be dispersed to distant habitats postfire via a wind vortex (He et al. 2009). The patterns of morphological divergence between the epicormic and lignotuberous populations of *B. attenuata* coincided with the differentiation of neutral genetic markers. The genetic populations are clustered into two groups: a lignotuberous and an epicormic cluster, suggesting the action of a nonrandom process. Geographic distance might drive the observed divergence in morphology and genetic differentiation. However, this is less likely the case for *B. attenuata*, as the geographic distances from the two epicormic populations near the suggested boundary to the center of the major cluster of resprouting types are similar (∼ 150 km), yet the *F*'_ST_ values for these two populations with respect to the lignotuberous and remaining epicormic populations are markedly different (0.28 vs. 0.03). It is therefore likely that the differentiated expression of postfire resprouting types in *B. attenuata* between epicormic and lignotuberous forms is the consequence of local genetic adaptations. Differentiation in neutral markers may have also been tied with such local genetic adaptation, likely as a consequence of long time independent adaptation and evolution of the two resprouting types in local environment.

Local adaptation to divergent local environments can lead to dramatic phenotypic differences between populations, which can be accomplished in the presence of ongoing gene flow. Genetic adaptation and phenotype plasticity are two ways in which organisms can adapt to local environments (Schlichting and Pigliucci 1998). Natural selection can act on the genetic variation present in a population over a number of generations, which results in a population being adapted to prevailing environmental conditions. Additionally, adaptive phenotypic change can occur within a generation, producing locally adapted phenotypes without genetic change (Doughty and Reznick 2004; Ghalambor et al. 2007; Latta et al. 2007). The marked morphological divergence between postfire resprouting types is unlikely to be a consequence of phenotypic plasticity, but rather a result of local genetic adaptation. The ability to form a lignotuber or epicormic bud-bearing meristem is controlled genetically. Lignotuber development and epicormic resprouting are also associated with changes in many morphological/ecophysiological traits that interact with each other. For example, interactions between lignotuber development, resource allocation, and the level of serotiny were observed in a lignotuberous population (Cowling and Lamont 1985); the development of a lignotuber is associated with the storage of a large amount of starch in woody roots (Bell and Ojeda 1999). Thus, postfire resprouting as a local adaptation stems from differences in the selection pressures created by different fire regimes and characteristics acting on heritable traits.

Locus BA-C112 presented higher genetic differentiation than expected, suggesting that alleles at this locus might have been affected by the action of selection among populations. Locus BA-C112 displays a correlation with epicormic resprouting, with one of its alleles being dominant in epicormic populations. This result may suggest a likely linkage of locus BA-C112 with genes involved in epicormic resprouting behavior in *B. attenuate*. However, it is also possible that locus BA-C112 is linked to other morphological or physiological trait in epicormic resprouting populations. This result echoes the concern that the choice of the so-called neutral markers be carefully evaluated in population genetics studies (Lazrek et al. 2009). Interestingly, the alleles of locus BA-C112 were not fixed in the lignotuberous population, which most likely implies that the genes responsible for the development of the lignotuber and related morphological and physiological characteristics are different from those associated with epicormic resprouting. However, strong genetic differentiation was revealed between populations exhibiting different resprouting modes. The close association of morphological divergence with genetic differentiation in neutral markers (even excluding the locus putatively associated with selection) suggests that the degree of genetic differentiation at neutral marker loci may be roughly indicative of the degree of differentiation among genes coding for functional traits (Merila and Crnokrak 2001).

### Evolutionary transition of resprouting types

The mode of postfire regeneration is a highly labile trait among banksias (Bond and Midgley 2003; He et al. 2011), and no phylogenetic signal was found to be associated with the biology of regeneration (Merwin et al. 2012). Wells (1969) proposed that resprouting after fire is ancestral to seeding in fire-prone environments, though a phylogeny-based analysis was not able to confirm such a hypothesis, at least in banksias (Bond and Midgley 2003; He et al. 2011). Through an anatomical analysis of early ontogenetic differences in axillary bud development, Verdaguer and Ojeda (2005) revealed an evolutionary transition from lignotuberous resprouting to nonsprouting (complete regeneration from seeds after fire) in *Erica* (Ericaceae). Based on an analysis involving approximate Bayesian computation, there was a possible evolutionary transition of the postfire resprouting type from epicormic to lignotuberous during range expansion in *B. attenuata*. It is worth noting that the evolutionary transition of the postfire resprouting type was concluded based on the detection of historical population bottleneck in lignotuberous populations and on the logic that the population that did not change size is more likely to be ancestral and to have kept ancestral characters. However, the bottleneck could have been caused by other factors than shift of fire-adaptation strategy, if less likely.

The analysis of the population history of *B. attenuata* suggests that the observed microevolutionary change in resprouting type is largely the result of directional natural selection underlying the genetic changes. Because epicormic populations of *B. attenuata* exist in the wetter part of the range of this species, which is also true for other banksias exhibiting these two postfire resprouting forms, it is likely that *B. attenuata* originated in a wet habitat and gradually expanded and adapted to drier environments with different fire regimes by switching to lignotuberous resprouting. Alternatively, it could have adapted *in situ* by switching to lignotuberous resprouting as its habitat became more arid and the associated fire regime changed in the Quaternary. ABC analysis revealed that the time of switch was at 23,800 years (19,600–28,800, 5–95% quantile), in consistent with age estimate (19,000–31,000 years) of sand dunes where the studied lignotuberous populations were situated (Krauss et al. 2006). The capacity to switch between postfire resprouting types, thus allowing adaptation to diverse fire regimes, is most likely the key factor explaining why *B. attenuata* is the most widespread species of the *Banksia* genus. Indeed, banksias that are able to resprout via multiple means are usually widespread (Lamont and Connell 1996).

Despite the evolutionary transition from epicormic to lignotuberous resprouting in *B. attenuata*, a significant shift in the genetic composition of the populations with distinctive resprouting syndromes is not evident. In fact, neither type exhibits unique alleles across all eleven examined microsatellite loci. Even at locus BA-C112, which is associated with the resprouting type, the two forms share the same array of alleles. Although no cross-pollination experiments were conducted between the two forms, no morphological/ecophysiological differences that could possibly have resulted in their reproductive isolation have been reported (George 1981; Taylor and Hopper 1988; Collins et al. 2008). This result suggests that the evolutionary response to environmental changes may be not necessarily dependent on the introduction of new genetic variation (Kelly et al. 2003; Jump et al. 2006) and that evolutionary transitions and local adaptations can arise from existing genetic variation (Barrett and Schluter 2008). Further interpretation of the conclusion needs to be cautious since it was drawn on genetic variation of eleven microsatellite loci. Further study examining broader genomic regions using next generation sequencing may be able to reveal a clearer landscape of genetic adaptation of postfire resprouting behaviors.
